# First insights into the molecular basis association between promoter polymorphisms of the *IL1B* gene and *Helicobacter pylori* infection in the Sudanese population: computational approach

**DOI:** 10.1186/s12866-020-02072-3

**Published:** 2021-01-07

**Authors:** Abeer Babiker Idris, Einas Babiker Idris, Amany Eltayib Ataelmanan, Ali Elbagir Ali Mohamed, Bashir M. Osman Arbab, El-Amin Mohamed Ibrahim, Mohamed A. Hassan

**Affiliations:** 1 Department of Medical Microbiology, Faculty of Medical Laboratory Sciences, University of Khartoum, Khartoum, Sudan; 2 Medical Laboratory Specialist, Department of Medical Microbiology, Rashid Medical Complex, Riyadh, Saudi Arabia; 3 Department of Medical Microbiology, Faculty of Medical Laboratory Sciences, University of Al-Gazirah, Wad Madani, Sudan; 4 Faculty of Medicine, University of Khartoum, Khartoum, Sudan; 5 Department of Internal Medicine, Modern Medical Centre, Khartoum, Sudan; 6 Department of Bioinformatics, Africa city of technology, Khartoum, Sudan; 7 Department of Bioinformatics, DETAGEN Genetic Diagnostics Center, Kayseri, Turkey; 8 Department of Translation Bioinformatics, Detavax Biotech, Kayseri, Turkey

**Keywords:** *H. pylori*, *IL1B*, 5′- region, In silico analysis, Sudan

## Abstract

**Background:**

*Helicobacter pylori* (*H. pylori*) infects nearly half of the world’s population with a variation in incidence among different geographic regions. Genetic variants in the promoter regions of the *IL1B* gene can affect cytokine expression and creates a condition of hypoacidity which favors the survival and colonization of *H. pylori.* Therefore, the aim of this study was to characterize the polymorphic sites in the 5′- region [−687_ + 297] of *IL1B* in *H. pylori* infection using in silico tools.

**Results:**

A total of five nucleotide variations were detected in the 5′-regulatory region [−687_ + 297] of *IL1B* which led to the addition or alteration of transcription factor binding sites (TFBSs) or composite regulatory elements (CEs). Genotyping of *IL1B* − 31 C > T revealed a significant association between -31 T and susceptibility to *H. pylori* infection in the studied population (*P* = 0.0363). Comparative analysis showed conservation rates of *IL1B* upstream [−368_ + 10] region above 70% in chimpanzee, rhesus monkey, a domesticated dog, cow and rat.

**Conclusions:**

In *H. pylori*-infected patients, three detected SNPs (− 338, − 155 and − 31) located in the *IL1B* promoter were predicted to alter TFBSs and CE, which might affect the gene expression. These in silico predictions provide insight for further experimental in vitro and in vivo studies of the regulation of *IL1B* expression and its relationship to *H. pylori* infection. However, the recognition of regulatory motifs by computer algorithms is fundamental for understanding gene expression patterns.

## Background

*Helicobacter pylori* (*H. pylori*) is a Gram-negative, spiral-shaped and microaerophilic bacterium that infects nearly half of the world’s population with a variation in incidence among different geographic regions [1, 2]. Epidemiological studies have indicated that the highest prevalence of *H. pylori* was found in Africa (79.1%), and the lowest prevalence was found in Northern America (37.1%) and Oceania (24.4%) with an overall global *H. pylori* prevalence of 44.3%, ranging from 50.8% in developing countries to 34.7% in developed countries [3–6]. The global annual recurrence rate of *H. pylori* was (4.3%) and it was found to be related to the human development index and prevalence of infection [3]. However, the clinical aspects of chronic infection with *H. pylori* vary from gastroduodenal inflammation and peptic ulceration to the most dangerous aspects, gastric carcinoma and mucosa-associated lymphoid tissue (MALT) lymphoma [7, 8]. Also, *H. pylori* may be implicated in several extra-gastric diseases such as iron deficiency anemia, idiopathic thrombocytopenic purpura, several dermatological disorders, hepatic encephalopathy, diabetes, and pulmonary and cardiovascular diseases [7, 9]. Indeed, the susceptibility to *H. pylori* infection and its diverse clinical presentation is determined by multiple factors, including heterogeneity of *H. pylori* strains and their virulence factors, environmental factors, and the host genetic background, especially those regarding polymorphisms in certain cytokines, gene regulation and their receptor antagonist genes [10–13]. One of these cytokines is the *interleukin 1-beta* (*IL1B*) gene.

*IL-1* family genes, spanning ~ 430 kb, cluster on chromosome 2q13–21 and consist of *IL-1A*, *IL-1B*, and *IL-1RN* genes which encode the pro-inflammatory cytokines IL-1α and IL-1β and the endogenous receptor antagonist IL-1ra, respectively [14]. IL-1β, the crucial cytokine in the gastrointestinal tract [15], has a variety of biological activities on a wide range of tissues and plays an important role in inflammatory, metabolic, physiologic, hematopoietic, and immunological processes (for a review, see references (16) and [17]). Because of the ability of IL-1β to inhibit gastric acid secretion, it may have a profound effect on the natural history of *H. pylori* infection by allowing expansion of *H. pylori* colonization from the gastric antrum to the corpus [15, 18–20]. On a molar basis, IL-1β is 100-fold more potent than proton pump inhibitors (PPIs) and 6000-fold more potent than H2 receptor antagonists [21].

IL-1β is expressed at high levels in myeloid cell lineages in response to tissue injury and microbial invasion [22, 23]. Also, many different types of cells, e.g. B cells, T cells, NK cells, dendritic cells, fibroblasts and epithelial cells, express this protein in response to a broad range of stimuli and under inflammatory conditions but at much lower level [24–26]. LPS-inducible IL1β expression is regulated by two regions: a proximal promoter that contains a TATA box and an upstream LPS-responsive enhancer (located between − 3757 and − 2729), which is also known as the upstream inducible sequence [27, 28]. In monocyte, this promoter is packaged into a highly accessible chromatin structure which is characterized by the constitutive association of PU.1 and C/ EBPβ, but the inducible association of RNA polymerase II [24, 29, 30]. The following multiple transcription factors that constitutively and inducibly associate with IL-1β regulatory regions have been identified: Spi-1/PU.1 (Spi1), NF-κB, C/EBPβ, AP-1, TBP, SSRP, or c-Jun and c-fos [29].

Genetic variations in the promoter region of genes encoding cytokines were shown to correlate with individual differences in the expression of respective cytokines which may influence the intensity of the inflammatory response and susceptibility to many diseases [31–35]. *IL-1B* gene has two allelic variants (CT; dbSNP: rs16944) and (TC; dbSNP: rs1143627) which are located at positions − 511 and − 31, respectively, in the promoter region. These SNPs have been proposed to be associated with the susceptibility to *H. pylori* infection; and *H. pylori*-related gastric cancer and peptic ulcer disease [36], but it is still a contradictory topic of debate. Many studies have been published analyzing the contribution of *IL1B* promoter polymorphisms to *H. pylori* susceptibility with conflicting results explained, in part, by ethnic differences [19, 32, 36]. In the present study, genomic DNA Sanger sequencing was applied to detect SNPs in the region [−687_ + 297] of *IL1B* in *H. pylori-*infected patients; and bioinformatics analyses were used to study whether these mutations would alter transcription factor binding sites (TFBSs). Further computational analysis was also made to investigate other potential regulatory elements in this region. Finally, comparative profiling was conducted to assess the conservation of these genetic variations in 11 species. However, to our knowledge, the association between promoter polymorphisms of the *IL1B* gene and *H. pylori* infection in the Sudanese population has not been studied. It is imaginable that individual differences in *H. pylori* susceptibility or individual differences in *H. pylori*-related disease severity are linked to genetically determined differences in *IL1B* production. Therefore, studying the regulation of *IL1B* gene expression is of great significance.

## Results

### Nucleotide variations in the 5′-regulatory region of the *IL1B* gene

In Sudanese *H. pylori* infected patients, a total of five nucleotide variations were detected in the 5′-regulatory region. Among which, three are bimodally mutated heterozygous SNPs, and they were newly discovered; the positions of these three SNPs are − 338, − 155 and + 38. The other two SNPs were rs16944 and rs1143627, see Table 1 for more illustration. The nucleotide sequences of the *IL1B* 5′- region [−687_ + 297] were deposited in the GenBank database under the following accession numbers: from MT767762 to MT767775.

**Table 1:** Nucleotide changes in the 5′-regulatory [−687_ + 297] region of the *IL1B* gene in *H. pylori*-infected patients

SNP	Event	SNP Locus	Chromosome Position	Serial Number (rs)
T > C (heterozygous)	Transition	+ 38	NG_008851.1:g.5039	–
C > T (heterozygous and homozygous)	Transition	-31	NG_008851.1:g.4970	rs1143627
G > C (heterozygous)	Transversion	−155	NG_008851.1:g.4844	–
A > T (heterozygous)	Transversion	−338	NG_008851.1:g.4666	–
T > C (heterozygous and homozygous)	Transition	−511	NG_008851.1:g.4490	rs16944

### In silico prediction of the *IL1B* promoter regions

Five types of promoter prediction programs were employed to predict the promoter regions of *the IL1B 5′- region* [−687_ + 297] and the results are presented in Table 2. The Promoter 2.0 Prediction Server predicted no promoter region. Neural Network Promoter Prediction (NNPP version 2.2) predicted three promoter regions, located at -328 bp, − 124 bp and + 1 bp relative to the *IL1B* translational start codon (transcript NM_000576.3), whose prediction scores were 0.97, 0.60 and 0.96, respectively. While Fprom, TSSG and TSSW programs predicted one promoter region, + 1 bp, which is the only region predicted by all used prediction programs.

**Table 2:** Overview of the computational predicted *IL1B* promoter regions for the respective prediction programs. The most predicted region is indicated in bold

Prediction program	Predicted promoter regions^a^
BDGP (NNPP version 2.2)^b^	−368 _ -319
	− 164 _ -115
	**−40 _ + 10**
Fprom	**+ 1**
Promoter 2.0 Prediction Server	no promoter
TSSG^c^	**+ 1**
TSSW^d^	**+ 1**

^a^All positions are given in base pairs relative to the translational* IL-1B* start codon (transcript NM_000576.3)

^b^Neural Network Promoter Prediction, Threshold 0.50

^c^Threshold for LDF- 4.00

^d^Thresholds for TATA+ promoters - 0.45, for TATA−/enhancers - 3.70

### In silico analysis of predicted *IL1B* promoter regions

**Fig. 1:**
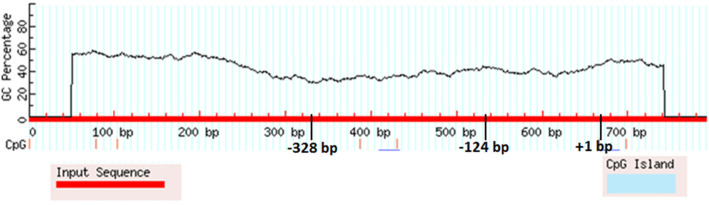
MethPrimer software prediction of no CpG islands in the in silico-predicted promoter regions

ENCODE data showed a high level of DNase I hypersensitivity, promoter associated histone modifications and transcription factor occupancy patterns at − 124 and + 1 bp promoter regions. While no Nuclease hypersensitivity around − 328 bp region. However, no CpG islands were detected in the predicted promoter regions (Fig. 1). Also, ENCODE data confirmed no presence of the CpG island in the predicted promoters.

#### Conservancy of the predicted promoter region [− 368_ + 10]

**Fig. 2:**
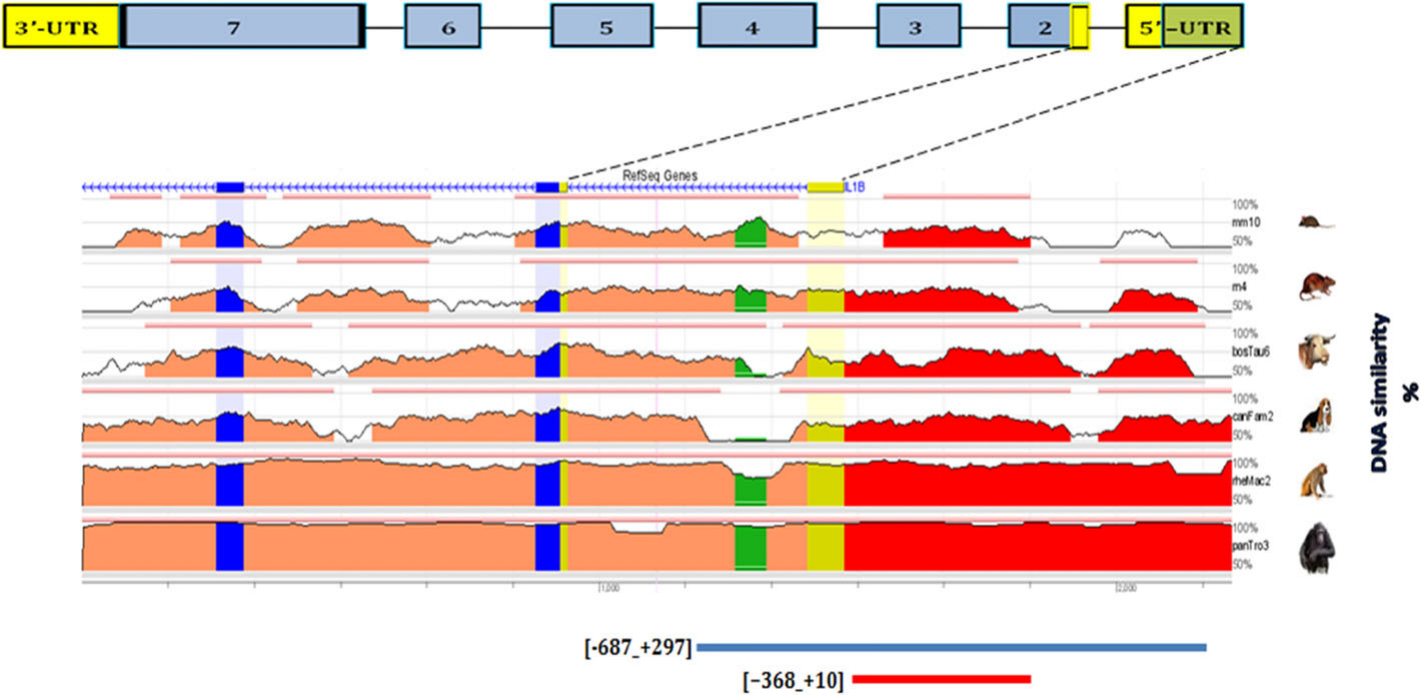
Shows conservation of the *IL1B* 5′-upstream region compared to the human sequence in region [−687_ + 297] (hg19 chr2:113594193–113,594,801). The height of the conservation plot at each position represents the number of nucleotides conserved in a window of 100 nucleotides centered on that position. The pink rectangles at the top of the plot represent the evolutionary conserved regions, which are defined as regions of 100 nucleotides with at least 70% identity. Blue boxes represent the *IL1B* exons, while yellow boxes indicate the *IL1B* 5′-UTR. Intragenic positions are highlighted in red, or in green when corresponding to transposable elements and simple repeats. Overview from the ECR Browser [37]

The ECR Browser revealed mammalian conservation for the [− 368_ + 10] region in chimpanzee (*Pan troglodytes* - pan-Tro2) (99.2%), rhesus monkey (*Macaca mulatta* - rheMac2) (93.4%), domesticated dog (*Canis lupus familiaris* - canFam2) (73%), cow (*Bos Taurus* - bosTau3) (71.1%), rat (*Rattus norvegicus* - rn4) (70.5%) and mouse (*Mus musculus* - mm9) (69.3%). But the region was not conserved in opossum (*Monodelphis domestica*), chicken (*Gallus gallus*), frog (*Xenopus laevis*), zebrafish (*Danio rerio*), fugu pufferfish (*Takifugu rubripes*), and spotted green pufferfish (Tetraodon nigrovoridis), see Fig. 2 for more illustration.

#### Prediction of TFBSs

**Fig. 3:**
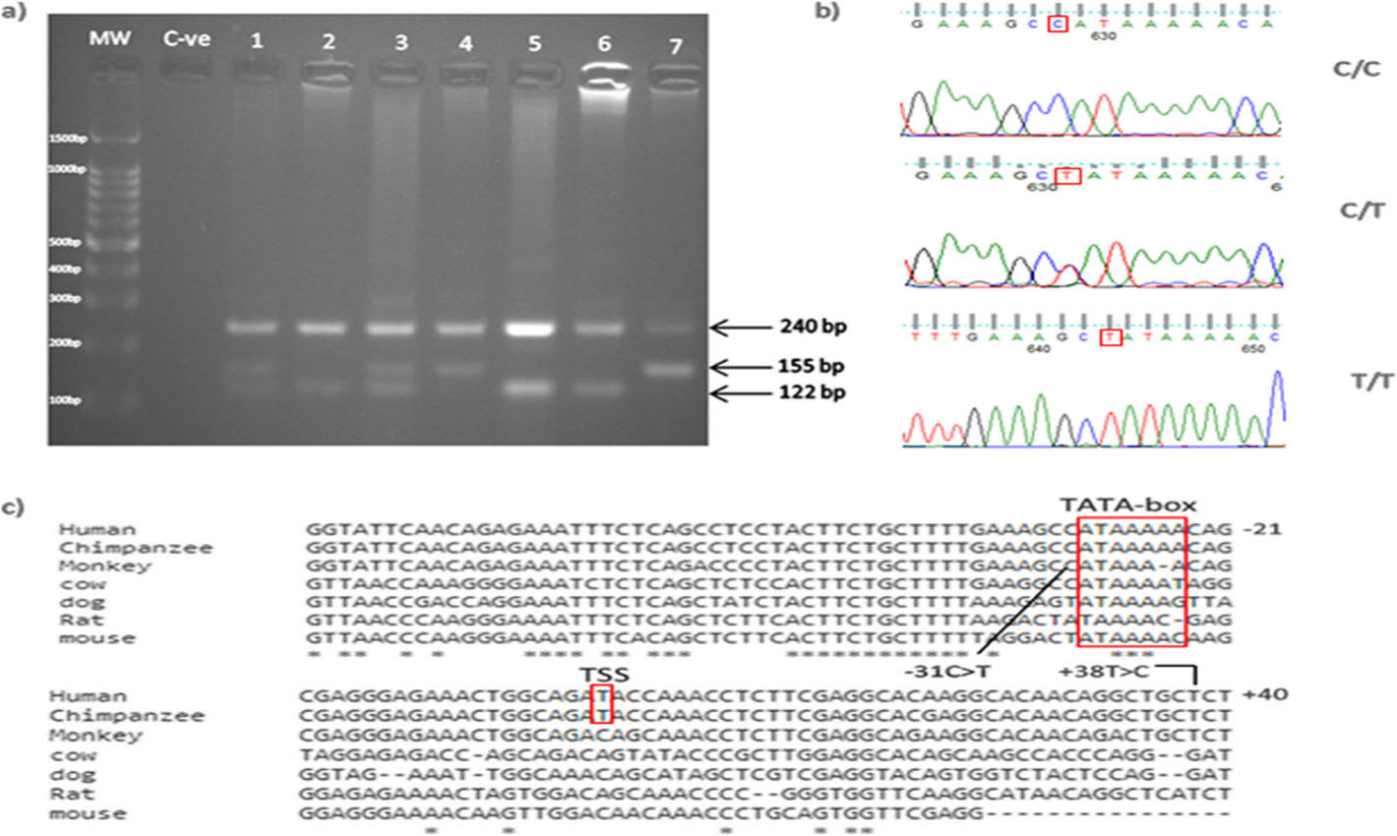
a) Illustrates PCR-CTPP products analyzed on a 2% agarose gel stained with ethidium bromide. Three genotypes can be seen. 1and 3 show 240 bp, 155 bp and 122 bp which indicate a heterozygous genotype. 2, 5 and 6 show 240 bp and 122 bp which indicate a homozygous T genotype. While 4 and 7 show 240 bp and 155 bp which indicate a homozygous C genotype. b) shows the sequencing result of the polymorphism by using Finch TV software. c) Illustrates the conservation of (TC; dbSNP: rs1143627) at position − 31 among different species

**Fig. 4:**
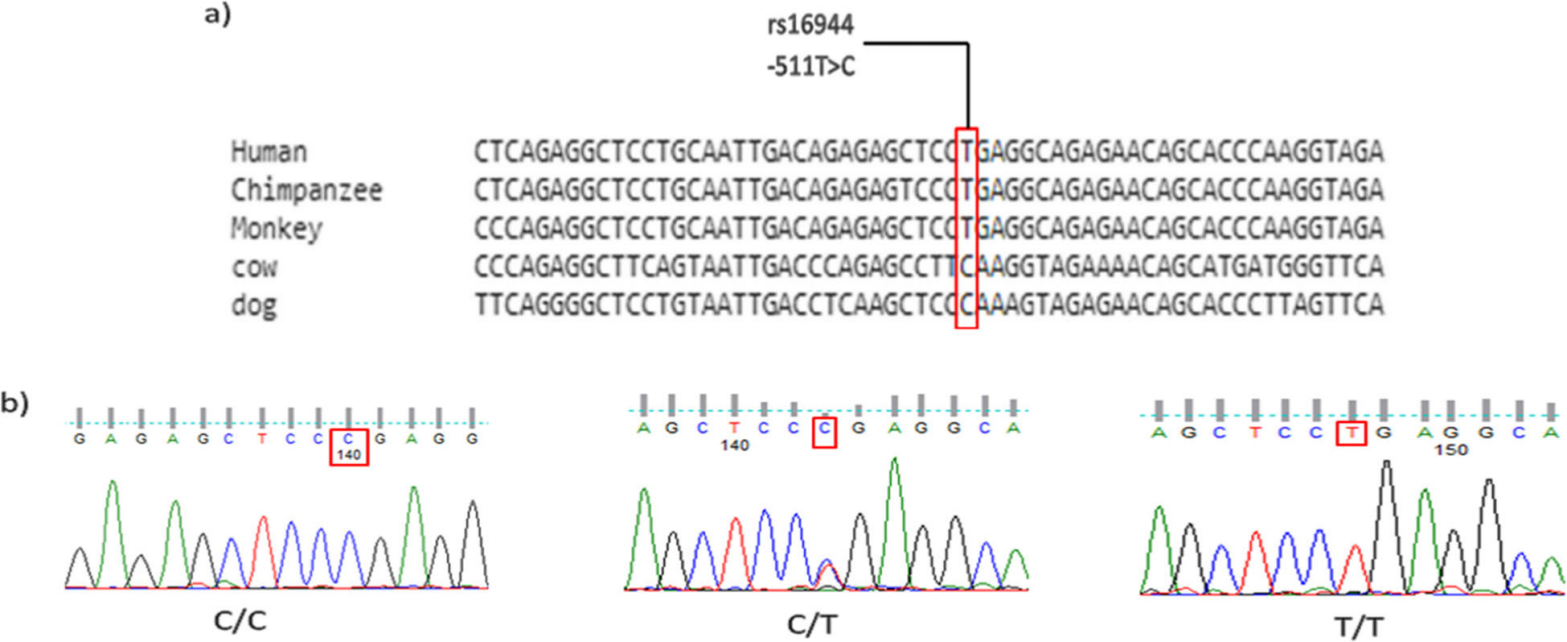
a) Conservation of (CT; dbSNP: rs16944) at position − 511 among various species. b) shows the chromatogram results of the polymorphism by using Finch TV software

**Fig. 5:**
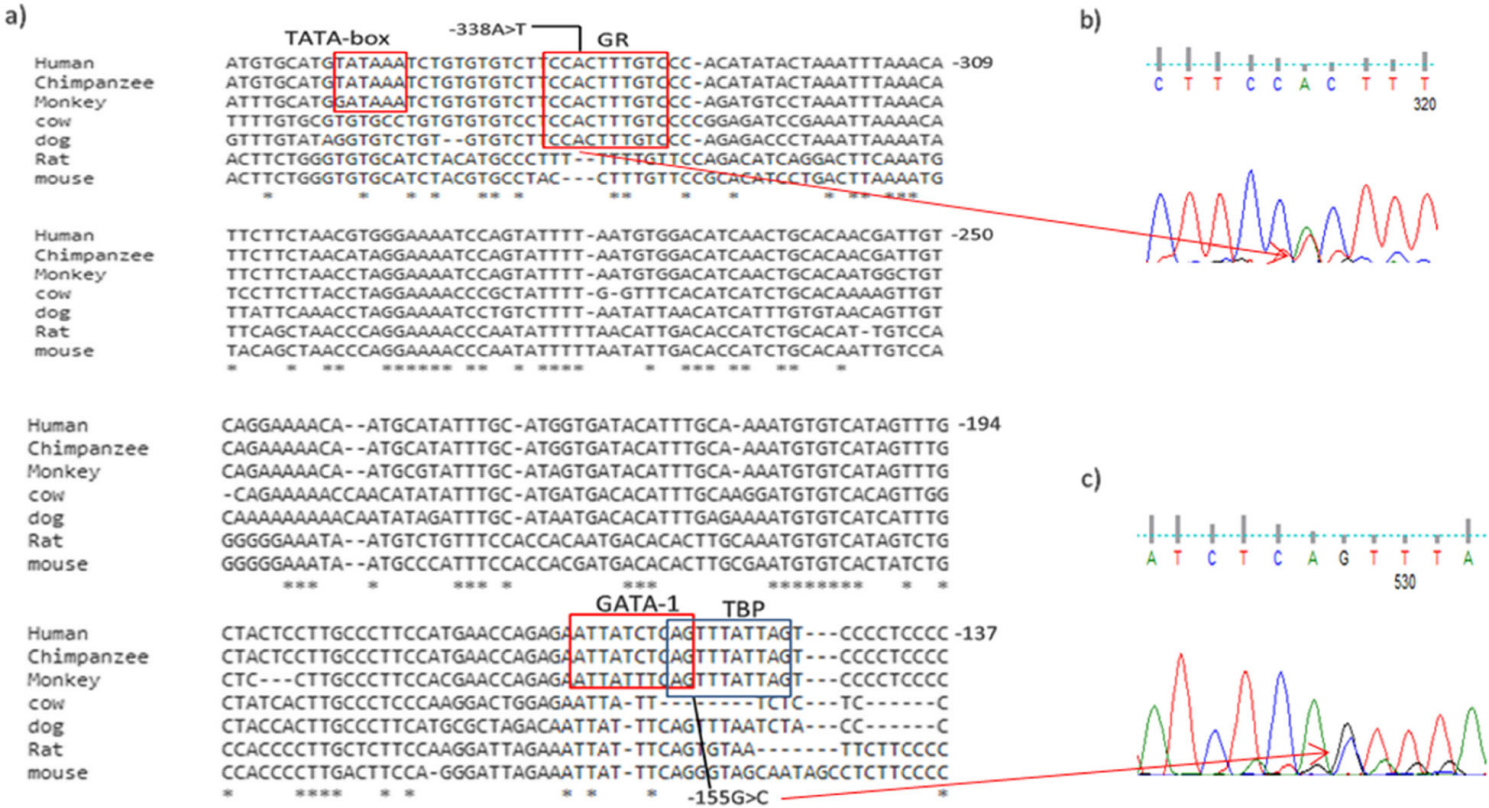
a) Overview on the mammalian conservation of -338A > T and -155G > C SNPs . The nucleotides are enumerated at each line on the right side. The in silico predicted TATA- and TFBSs are marked in boxes. b) and c) Shows the chromatogram results of the polymorphisms by using Finch TV software

In this study, five programs were used to predict TFBSs and to insure proper analysis we only selected factors that are predicted by three out of the five programs or the factors predicted by two programs but verified in the literature. The five prediction programs reported multiple putative TFBSs within the [−368_ + 10] region, see Table 3 and Figs. 3, 4 and 5. However, screening of this region, by using the NCBI SNP databases (dbSNPs), revealed the presence of 9 SNPs upstream of the *IL1B* core promoter region which are shown in Table 4. The ECR Browser and NCBI BLASTn showed the conservation of these SNPs in chimpanzee, rhesus monkey, cow and dog. Mulan revealed multiple TFBSs to be located at rs749558279, rs140623868 and -338A > T. The overview of conserved TFBSs predicted by Mulan to be conserved (100%) between human, chimpanzee, rhesus monkey, cow and domesticated dog is summarized in Table 4.

**Table 3:** Summary of the in silico predicted TFBSs for the [−368_ + 10] region

Transcription factor	Position^a^		SNP in binding site	Software				
	Start	End		AliBaba2.1	Alggen Promo	Tfsitescan	TF-Binding	GPMiner
GR	− 345	− 327	rs140623868 NG_008851.1:g.4666 A > T^b^	X	X	X	X	
NF-kappaB	−297	− 288		X	X	X	X	
c-Myb	− 269	− 260	rs769027934		X	X	X	
Oct-1	− 233	− 225		X		X	X	
GATA-1	− 227	− 214	rs749558279	X	X	X	X	X
GR	− 207	− 202			X	X	X	
GATA-1	−164	−155	rs866837107 NG_008851.1:g.4844 G > C^b^	X	X	X		
Sp1	− 149	− 132	rs74579367	X		X		
AP-2	− 148	− 137				X	X	
MAZ	− 147	−137			X	X		X
NF-Y	− 126	−114			X	X	X	
NF-AT	− 116	− 105			X			X
Spi-1/PU.1	−115	−97		X		X		
STAT1	− 108	− 101			X		X	X
IRF	− 106	−99			X	X		
C/EBPalpha	−94	−83			X		X	X
NF-kappaB	−70	− 61	rs4986962	X			X	
HSTF/HSF	−69	−60		X			X	
C/EBP	−67	−58		X			X	
Spi-1/PU.1	−50	−39		X		X		
TBP	−31	−25	rs1143627^b^		X		X	X
c-Myb	−14	−5			X		X	X
NF-1	−13	−4		X	X		X	
GATA-1	−7	6	rs529869449		X	X	X	X

^a^Positions are given relative to the translational *IL1B* start codon (transcript NM_000576.3)

^b^SNPs observed in this study

**Table 4:** Conserved TFBSs predicted by Mulan (https://mulan.dcode.org/) to be conserved (100%) between human, chimpanzee, rhesus monkey, cow and domesticated dog at the [−368_ + 10] region. The most important transcription factor Sp1 for *IL1B* transcription was not included in this list

Transcription Factor	Binding sequence	Position^a^		Conserved SNPs
		Start	End	
HNF4_01	TCTTCCACTTTGTCCCACA	− 344	− 326	rs140623868 [− 335] -338A > T^b^
DR1_Q3	TCCACTTTGTCCC	− 341	− 329	
PPAR_DR1_Q2	TCCACTTTGTCCC	−341	−329	
NFKAPPAB65_01	GGAAAATCCA	− 295	− 286	
OCT1_01	TGCATATTTGCATGGTGAT	− 236	− 220	rs749558279 [− 224]
OCT1_Q5_01	ATATTTGCATG	− 235	−225	
OCT_Q6	ATATTTGCATG	−235	−225	
OCT1_B	TATTTGCATG	− 234	−225	
AP1_Q2_01	CAAAATGTGTCA	− 212	− 201	
ZTA_Q2	TGTGTCATAGTTT	−207	− 196	
CEBPDELTA_Q6	GATTGTGAAATC	−93	−82	
BARBIE_01	ACTTCTGCTTTTGAA	−49	−35	

^a^All positions are given in base pairs relative to the translational IL1B start codon (transcript NM_000576.3)

^b^Observed in this study

#### Prediction of CEs

MatrixCatch was used to find known regulatory elements (both single sites and pairs) which were verified experimentally. Also, it found novel regulatory elements by computational comparison but without experimental verification on functionality. These elements were found by using similarity to known ones in a library of CE models [38]. The summary of predicted CEs by MatrixCatch is presented in Table 5.

**Table 5:** Summary of the in silico predicted CE for the [−368_ + 10] region

CE	MatrixName for a 1st element	S_1_	Distance in between	MatrixName for a 2nd element	S_2_	Position^a^	orientation	CS	*P*-Value	Sequence
CE00047	V$POU1F1_Q6	0.935	−1	V$OCT1_04	0.949	− 239	–	−0.004	7.72e-06	ATGCATATTTGCATGGTGATAC
CE00058	V$NFKB_Q6_01	0.937	5	V$HMGIY_Q6	0.995	− 300	+	−0.46	1.22e-05	ACGTGGGAAAAT
CE00158	V$OCT_C	0.907	10	V$AP1_01	0.869	−236	–	−0.232	1.88e-04	CATATTTGCATGGTGATACATTT
CE00058	V$NFKB_Q6_01	0.791	6	V$HMGIY_Q6	0.995	− 301	+	−0.181	1.05e-03	AACGTGGGAAAAT
CE00186	V$ETS_Q6	0.936	13	V$CEBPA_01	0.942	−107	–	−1.049	6.55e-03	CTTTCCTTTaactTGATTGTGAAATCA
CE00249	V$IRF_Q6	0.924	5	V$PU1_Q6	0.839	− 111	+	−0.314	1.01e-02	TCCCCTTTCCTTT
CE00078	V$GR_Q6	0.914	39	V$CEBPB_02	0.799	− 377	+	0.049	2.37e-02	GAAGAAAAGTATGTGCATGTataaatctgtgtgtcttccACTTTGTCCCACAT
CE00186	V$ETS_Q6	0.896	20	V$CEBPA_01	0.942	− 120	–	−0.793	2.73e-02	TTTTCCCCTttcctttaactTGATTGTGAAATCA
CE00135	V$ETS_Q6	0.781	25	V$MYB_Q5_01	0.974	− 292	–	0.002	4.31e-02	AAATCCAGTattttaatgtggacatCAACTGCAC

*CE* Composite regulatory element, *S*_*1,2*_ PWM scores for the first and second elements, *CS* Composite score

^a^Beginning of the element relative to TSS

### Allele and genotype frequencies of *IL1B-*31 and susceptibility to *H. pylori* infection

Sixty-one patients and 61 uninfected controls were successfully genotyped for the *IL1B*-31 C/T polymorphism (Fig. 4). The frequency of allele T and *IL1B*-31 T/C + T/T genotypes were significantly higher in *H. pylori*-infected individuals compared to uninfected controls (36.89% versus 23.77%, *P* = 0.0363 and 54.1% versus 34.43%, *P* = 0.0445, resp.). The allelic and genotype distributions of *IL1B*-31 C/T polymorphism followed those expected in Hardy-Weinberg equilibrium (HWE) for control population (*P* = 0.1366) (Table 6).

**Table 6:** Allele and genotype frequencies of *IL1B*-31 polymorphism among *H. pylori* infected and uninfected subjects, and their contributions to *H. pylori* infection

	*H. pylori* (+ve) *n* = 61	*H. pylori* (−ve) *n* = 61	OR (95% CI)^*^
Allele frequency			
*IL1B-31-C*	77 (63.11%)	93 (76.23%)	0.5336 (0.3060–0.9304)
*IL1B-31-T*	45 (36.89%)	29 (23.77%)	
P-value	**0.0363**		
Genotype frequency			
C/C	28 (45.90%)	40 (65.57%)	1 (reference)
T/C	21 (34.41%)	13 (21.31%)	0.4333 (0.1864–1.008)
T/T	12 (19.67%)	8 (13.12%)	0.4667 (0.1688–1.290)
T/C + T/T	33 (54.1%)	21 (34.43%)	0.4455 (0.2147–0.9243)
P-value	**0.0445**		

^*^OR, odds ratio; 95%CI, 95% confidence interval

## Discussion

Genetic variants in the promoter region of *IL1B* gene can affect cytokine expression and create a condition of hypoacidity which favors the survival and colonization of *H. pylori* [15, 36]. In the present study, we functionally analyzed SNPs in the *IL1B* 5′-region [−687_ + 297] of Sudanese patients infected with *H. pylori* and developed divergent clinical outcomes. We observed three novel mutations (− 338, −155 and + 38) and interestingly, two of them (− 338 and − 155) were located at in silico-predicted promoter regions. Thus, these mutations might play a role in regulating the expression of *IL1B*. In this study, the computational analysis predicted three promoter regions at − 328, − 124 and + 1, but two of them (− 328 and − 124) were only predicted by the NNPP algorithm that uses neural networks (NNs). NNs have been applied for promoter prediction since 1991 [39]. The study conducted by Liu and States et al. compared different available prediction techniques during the development of their own technique, and showed that although NNPP2.2 is competitive with several other freely available techniques, the technique suffers from a high level of false positives [40]. However, many studies have used this technique for promoter predictions such as [41–44]. Clearly, the result obtained by this technique or other in silico tools cannot substitute for the experimental proofs but it can provide a direction or guidance for such experiments to validate computational predictions.

Nuclease hypersensitivity and histone modifications are characteristic for cis-regulatory regions such as promoters. The ENCODE data shows these hallmarks to be present in the putative promoter region at the + 1 bp region. The upstream region around − 124 bp showed some of these characteristics, although to a lesser degree, while the region around − 328 bp showed only histone marks [45–47]. Also, no CpG islands were detected in predicted promoter regions, however, most promoters with a TATA box do not have high GC content [48]. In silico comparative analysis showed the [−368_ + 10] region to be mammalian conserved, with conservation rates above 70% in chimpanzee, rhesus monkey, a domesticated dog, cow and rat. This conservation might indicate a possible regulatory role for this region (Fig. 2). But the region was not conserved in opossum, chicken, frog, zebrafish, fugu pufferfish, and spotted green pufferfish; it is possible that the regulation of *IL1B* in these species is controlled by a different mechanism or pathway.

Regulation of gene transcription depends on the interaction between TFs and TFBSs. Any changes in these sites may develop significant effects on the binding of TFs to regulatory sequences and then the expression products of genes [44, 49, 50]. In this study, an in silico-based prediction analysis using different algorithms indicated that the transcription factors NF, C/EBP, Spi-1/PU.1, NF-kappaB, AP-1, TBP, IRFs and STAT, c-Myb and GATA-1 are involved in the regulation of *IL1B* gene expression and have the potential to bind in the polymorphic regions (Table 3). This indication is in agreement with the results of previous studies [26, 51, 52]. The two novel SNPs located in the in silico-predicted promoter region led to the addition or alteration of the TFBSs. As illustrated in Table 7, − 338 (A > T) polymorphism resulted in the alteration of GR to PU.1 and the − 155(G > C) polymorphism led to an addition of a C/EBPbeta. T allele in position − 31 instead of C allele resulted in an addition of RSRFC4 protein. This finding is partially in agreement with experiments that assessed allele-specific oligonucleotides for − 31. The experiments reported that there were one or more TFs resulting in a fivefold increase in DNA binding on the *IL1B*-31 T oligonucleotide after LPS stimulation. These TFs may be unable to interact with the C-bearing *IL1B*-31 allele to form the transcription initiation complex [53].

**Table 7:** Variations in transcription factors before and after nucleotide changes (− 338, − 155 and − 31) in the *IL1B* gene by using AliBaba2.1 software

SNP Site	Base Group	TF	TFBSs Sequence	TF Position
−338	A	GCR1	CTTCC**A**CTTT	− 343 _ -334
		GR	C**A**CTTTGTCC	− 339 _ -330
	T	PU.1	CTTCC**T**CTTT	− 343 _ -334
		GCR1		
−155	G	Zen-1	ATTATCTCA**G**	−164 _ -155
		GATA-1	TTATCTCA**G**	− 163 _ -154
	C	C/EBPbeta	ATTATCTCA**C**	−164 _ -155
		Zen-1		
		GATA-1	TTATCTCA**C**T	−163 _ -154
−31	C	–	–	–
	T	RSRFC4	GC**T**ATAAAAA	−33 _ -24

The extensively studied SNPs in relation to *H. pylori* infection (−31 and − 511) were also detected in our patients. We observed a significant association between -31 T and susceptibility to *H. pylori* infection in the studied population (P = 0.0363). This result is in concordance with a number of studies conducted in different ethnic groups that showed an association between *IL1B*-31 T and *H. pylori* infection [53–58]. Also, there are some studies that found a negative association [33, 58, 59]. This variation could be due to differences in genetic backgrounds of the studied population, the method of genotyping and sample size [36, 60]. Interestingly, we found that the T-511C SNP was not located in the in silico-predicted promoter regions, hence it could not affect the expression of *IL1B*. While − 31 which involves a TATA-box could directly affect the induction of *IL1B*. These findings are in agreement with the result obtained by Al-Omer et al. that the − 31 polymorphism was markedly affecting DNA-protein interactions in vitro while − 511 does not alter in vitro protein secretion and its effect may be mediated by linkage disequilibrium (LD) with − 31 [53]. Also, R Kimura et al. found that the expression of the -31 T allele was 2.2 times of the -31C allele and this higher transcription efficiency may correspond to the fact that C-31 T is located in a TATA box [61]. In contrast, other observations of IL-1β production have suggested that there was no significant association between the known allelisms in the *IL-1B* gene and IL-1β induction in vitro and that the -31C was the higher expressing allele in vivo [61–63]. However, the production of IL1β is affected by several factors besides gene polymorphisms such as epigenetic conditions and other genetic backgrounds. To exclude the influence of trans-acting factors which are able to confound the effects of the polymorphisms, the allele-specific transcript quantification coupled with haplotype analysis [61, 64] is recommended to identify the cis-acting effect of T-511C polymorphism and our novel detected polymorphisms (− 338 and − 155) on the *IL1B* transcription and susceptibility to multifactorial diseases including *H. pylori* infection.

However, recognition of regulatory motifs by computer algorithms is fundamental for understanding gene expression patterns, as well as, cell specificity and development [49]. Identifying SNPs that might be a genetic modifiers in *IL1B* gene may be valuable in preventive, diagnostic, and therapeutic strategies against the incidence and progression of *H. pylori* infection. This study revealed three nucleotide variations in the *IL1B* 5′-region which possibly lead to modification of transcriptional regulation in *H. pylori* infection, however, this conclusion requires further in vitro and in vivo validation in subsequent studies.

## Conclusions

In *H. pylori*-infected patients, three detected SNPs located in the *IL1B* promoter were predicted to alter CEs and TFBSs, which might affect the gene expression. This computational analysis provide insight for further experimental in vitro and in vivo studies of the regulation of *IL1B* expression and its relationship to *H. pylori* infection. However, recognition of regulatory motifs by computer algorithms is fundamental for understanding gene expression patterns.

## Methods

### Study methodology

**Fig. 6:**
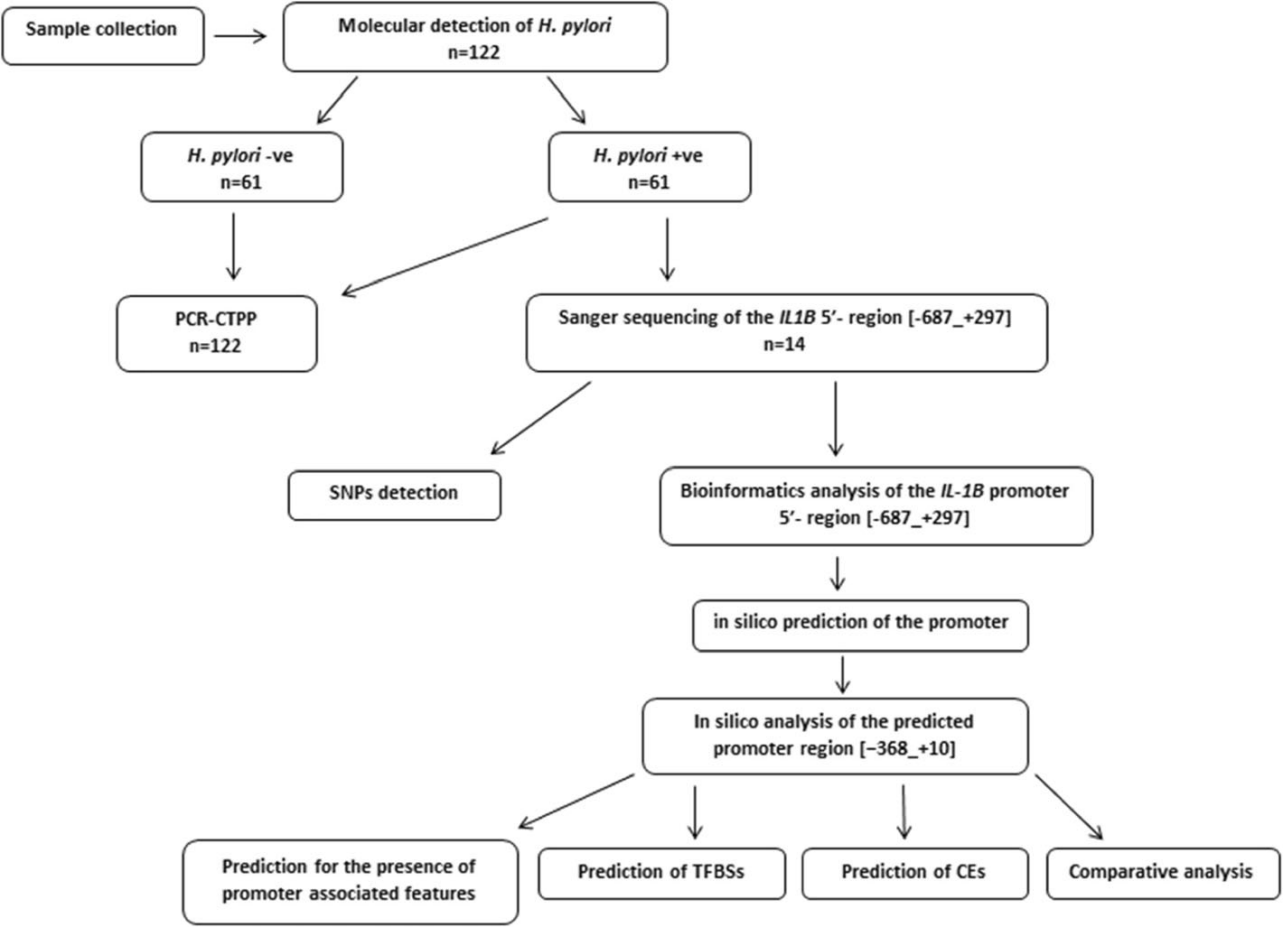
Schematic representation of the methodology

In this study, genomic DNA Sanger sequencing was used to detect SNPs in the region [−687_ + 297] of *IL1B* in 14 *H. pylori*-infected patients. Then, computational analyses of the *IL-1B* promoter region [−687_ + 297] were applied in two steps: 1) in silico prediction of the promoter region and 2) in silico analysis of the predicted promoter region [−368_ + 10]. Furthermore, genotyping of *IL1B*-31 C > T polymorphism was performed using PCR-CTPP in 122 participants to study its association with the susceptibility to *H. pylori* infection in the Sudanese population. The methodology followed in this study is described in Fig. 6.

### Study setting and study population

This study was carried out at public and private hospitals in Khartoum state. The hospitals included Ibin Sina specialized hospital, Soba teaching hospital, Modern Medical Centre and Al Faisal Specialized Hospital. Sample size was calculated using Epi Info software version 7 [65, 66]. The matched case-control formula was selected assuming 95% confidence level, 80% power of study, 1 ratio of control to case, 15% of controls exposed, 3.36 odds ratio and 37.2% of cases exposed. Based on the sample size calculation, a total of 122 individuals were recruited for this study.

The 122 participants had been referred for endoscopy. Out of that, 15 had gastric cancer, 27 had peptic ulceration, 61 had gastroduodenal inflammation, 10 had esophageal diseases, while nine showed normal upper gastroduodenoscopic features. The diagnosis of gastroduodenal diseases had been made by an experienced gastroenterologist during the upper gastrointestinal (GI) endoscopy procedure. While gastric cancer was diagnosed based on histology. Participants’ demographic and clinical data were obtained by a structured questionnaire, personal interviews, and a review of case records. The selection criteria included the Sudanese population from both sexes, no antibiotic or non-steroidal anti-inflammatory drugs (NSAIDs) uses. All the participants were informed with the objectives and purposes of the study and the written informed consents were taken. The demographic characteristics of participants is presented in Table 8.

**Table 8:** Demographic characteristic of participants

Variables	Total *n* = 122	*H. pylori* (+ve) n = 61	*H. pylori* (−ve) *n* = 61	P-value
Age years ± Std. Deviation (range)	44.37 ± 17.48 (15–89)	44.00 ± 16.99 (15–85)	44.74 ± 18.10 (17–89)	0.9184
Gender				
Male	72 (59.02%)	36 (50%)	36 (50%)	1.0000
Female	50 (40.98%)	25 (50%)	25 (50%)	
Residence				
Urban	54 (44.26%)	24 (44.44%)	30 (55.56%)	0.3622
Rural	68 (55.74%)	37 (54.41%)	31 (45.59%)	

### DNA extraction

Gastric biopsies were collected in 400 μl phosphate buffer saline (PBS). For histological examination, the biopsies were transported in formalin. DNA extraction was carried out by using innuPREP DNA Mini Kit (analytikjena AG, Germany) according to the protocol given by the manufacturer, as previously described in [67].

### PCR amplification of specific *16S rRNA* of *H. pylori*

The specific *16S rRNA* gene of *H. pylori* was amplified by using the following primers (primers: F:5′-GCGCAATCAGCGTCAGGTAATG-3′) (R:5′-GCTAAGAGAGCAGCCTATGTCC-3′) [68]. The PCR condition was previously described [69].

### PCR amplification and sequencing of the *IL1B* promoter region

The *IL-1B*-511 and − 31, promoter polymorphisms, were amplified using the following primers: F:5′- CATCCATGAGATTGGCTAG-3′ and R:5′- AGCACCTAGTTGTAAGGAAG-3′ [70]. The cycling conditions were an initial denaturation at 94 °C for 5 min, followed by 35 cycles of 94 °C for 1 min, 60 °C for 1 min and 72 °C for 1 min, with a final extension at 72 °C for 7 min. The amplified PCR product is 800 bp and was located between − 687 bp upstream and + 297 bp downstream of the *IL-1B* gene.

Out of 14 PCR products of *H. pylori*-infected subjects, which have the clearest bands, were sent for DNA purification and Sanger dideoxy sequencing. Both DNA strands were sequenced commercially by Macrogen Inc., Korea.

### Sequence analysis and SNPs detection

The sequencing results, two chromatograms for each patient (forward and reverse), were visualized, checked for quality, and analyzed using the Finch TV program version 1.4.0 [71]. The nucleotide Basic Local Alignment Search Tool (BLASTn; https://blast.ncbi.nlm.nih.gov/) was used to assess nucleotide sequence similarities [72].

To determine the SNPs in the *IL-1B* promoter region, multiple sequence alignment (MSA) for tested sequences with a reference sequence (NG_008851) were performed by using BioEdit software [73].

### Bioinformatics analysis of the *IL-1B* promoter region in *H. pylori*-infected subjects

#### In silico prediction of the promoter

The crucial element for initiating and regulating messenger RNA transcription is the promoter sequence which is generally located in the 5′ upstream region of a structural gene [44]. Promoters have complex and specific architecture, and contain multiple TFs involved in specific regulation of transcription [74]. Different features of a promoter region may have different power for promoter identification [49], therefore, we applied a variety of programs for prediction of promoter regions in order to obtain accurate results for subsequent experimental proof. These programs include: (1) Promoter 2.0 Prediction Server (http://www.cbs.dtu.dk/) which takes advantage of a combination of elements similar to neural networks and genetic algorithms to recognize a set of discrete sub-patterns with variable separation as one pattern: a promoter [75]; (2) Neural Network Promoter Prediction (NNPP2.2) (http://www.fruitfly.org/) which applying multiple hidden layers and time-delay neural networks (TDNNs) for promoter annotation [76]; (3) TSSW (http://softberry.com/) that uses functional motifs from the Wingender et al. database [77] and linear discriminant function combining characteristics describing function motifs and oligonucleotide composition of these sites [78]; (4) TSSG program (http://softberry.com/) program that uses the same approach of TSSW but the TFD database of functional motifs [79]; (5) Fprom program (http://softberry.com/) which is TSSG variant with different learning set of promoter sequences [49].

#### In silico analysis of the predicted promoter region

##### Assessment for the presence of promoter associated features

In silico predicted promoter region was additionally assessed for the presence of promoter associated features, including promoter-associated histone marks, broad chromatin state segmentation, transcription factor ChIP-seq, and DNase I hypersensitivity clusters, using the ENCODE data (https://epd.epfl.ch/cgi-bin/get_doc?db=hgEpdNew&format=genome&entry=IL1B_1) [45–47].

##### Prediction of CpG Islands

A CpG island is often regarded as a marker for the initiation of gene expression. It is a segment of DNA with high GC and CpG dinucleotide contents which is located in the 5′ UTR (untranslated regions) of genes. In this study, MethPrimer [44, 80] and GpC finder software (http://www.softberry.com/berry.phtml?topic=cpgfinder&group=programs&subgroup=promoter) were employed to predict CpG islands in the promoter. CpG finder is intended to search for CpG islands in sequences, while MethPrimer is developed to design PCR primers for methylation mapping and primers are picked around the predicted CpG islands. CpG islands are predicted by using a simple sliding window algorithm to examine the GC content and the ratio observed/expected (Obs/Exp) across the sequence. The search parameter values for the software were CpG island length > 200 bp, CG% > 50%, and Obs/Exp > 0.6.

##### Prediction of transcription factor binding sites (TFBSs)

One of the important steps in the chain of promoter analytical events is the prediction of the potentially functional TFBSs. Protein binding sites in a promoter represent the most important elements and the corresponding proteins are called transcription factors (TFs). In this step, the promoter region was analyzed for possible TFBSs using five prediction software. (1) Alggen Promo (http://alggen.lsi.upc.es/cgi-bin/promo_v3/) in which positional weight matrices (PWM) are constructed from known binding sites extracted from TRANSFAC [38] and used for the identification of potential binding sites in sequences [81, 82]. (2) AliBaBa2 (http://www.gene-regulation.com/) which works based on the assumption that each binding site has an unknown context that determines its sequence and this leads to a construct of specific matrices for each sequence we are analyzing. And to do so a context-specific process starting at a dataset of known binding sites and ending with the identification of a potential new binding site [83]. (3) Gene Promoter Miner (GPMiner) (http://GPMiner.mbc.nctu.edu.tw/) which is an integrated system that identifies promoter regions, regulatory elements and DNA stability by incorporating the support vector machine (SVM) with nucleotide composition features, over-represented hexamer nucleotides, and DNA stability. For predicting TFBSs, MATCH tool [84] was utilized to scan TFBSs in an input sequence using the TF binding profiles from TRANSFAC public release version 7.0 [85] and JASPAR [86, 87]. (4) TF-Bind (http://tfbind.hgc.jp/) which uses positional weight matrices (PWMs) and Bucher’s calculating method [88] to calculate the matching score between an input sequence and a set of known TF binding sites. To estimate TF binding sites, a robust cut-off value determining algorithm was proposed using the background rate estimated on non-promoters sequences [89]. (5) Tfsitescan (http://www.ifti.org) which is an object-oriented transcription factors database (ooTFD)-retrieval tool that is used for transcription factors sites analyses. It constructs an image-map in association with sequence analysis results which is linked to individual sites entries [90].

##### Prediction of composite regulatory elements (CEs)

CE is the minimal functional unit, which can provide combinatorial transcriptional regulation of gene expression. Structurally, a CE consists of two closely located DNA binding sites (BSs) for distinct transcription factors. But its regulatory function is qualitatively different from regulation effects of either individual DNA binding sites. In this study, we identified the composite regulatory elements in our region by using MatrixCatch algorithm (http://gnaweb.helmholtz-hzi.de/cgi-bin/MCatch/MatrixCatch.pl). The basic idea of MatrixCatch is to recruit data collected for respective binding sites separately from each other in order to complement the lack of knowledge on sequence variation of each DNA BS in CEs, and such information is compiled in position weight matrices (PWMs). The CE model consists of two PWMs, as well as their minimal scores, relative orientation and distance. Moreover, MatrixCatch is supplied with a library of 265 matrix models used for recognition which represents the widest scope of known CEs available to date [91].

##### Comparative analysis

Promoter region was analyzed for possible conservation using the ECR Browser (http://ecrbrowser.dcode.org) [37], NCBI BLASTn (http://blast.ncbi.nlm.nih.gov/Blast.cgi) and ClustalW (https://www.genome.jp/tools-bin/clustalw). Conservation was assessed in 11 species: chimpanzee (*Pan troglodytes*), rhesus monkey (Macacamulatta), mouse (*Mus musculus*), rat (Rattusnorvegicus), dog (Canisfamiliaris), cow (Bostaurus), opossum (Monodelphisdomestica), chicken (*Gallus gallus*), frog (Xenopuslaevis), zebrafish (*Danio rerio*), fugu pufferfish (Takifugurubripes), and spotted green pufferfish (Tetraodon nigrovoridis).

Also, conservation of SNPs was evaluated and the possible conservation of TFBSs at these SNP locations was screened with Multiple-sequence local alignment and visualization (Mulan) search engine (https://mulan.dcode.org/) [92].

### Detection of the *IL-1B*-31 C/T polymorphism using PCR with confronting two-pair primer (PCR-CTPP)

For detection of the *IL-1B*-31 polymorphism, PCR-CTPP was applied. The primers for the C allele were (F:5′-ACT TCT GCT TTT GAA GGC C-3′) and (R:5′-TAG CAC CTA GTT GTA AGG A-3′); and those for the T allele were (F:5′-AGA AGC TTC CAC CAA TAC T-3′) and (R:5′-CTC CCT CGC TGT TTT TAT A-3′) [93]. One μl of extracted DNA was used in a 25 μl reaction mixture with a prepared Maxime PCR PreMix Kit (*i*-Taq) (iNtRON BIOTECHNOLOGY, Seongnam, Korea), 23 μl of de-ionized sterile water, 0.25 μl of each primer. PCR conditions were as follow: 5 min of initial denaturation at 94 °C, followed by 25 cycles of 1 min at 94 °C, 1 min at 54 °C, and 1 min at 72 °C, and a 5 min final incubation at 72 °C. The PCR products were visualized by electrophoresis on a 2% agarose gel stained with ethidium bromide. Genotyping was performed as follows; 240, 155 bp for CC genotype, 240, 155, 122 bp for CT genotype, and 240, 122 bp for TT genotype [93].

### Statistical analysis

Deviations from Hardy-Weinberg equilibrium in control were examined by *χ*^*2*^ test. According to prevalence *of H. pylori* infection, differences in distribution by age were assessed by Mann-Whitney test, while differences in distribution by categorical variables were examined by *χ*^*2*^ test or *Fisher’s* test. Odds ratios (ORs) were calculated and reported within the 95% confidence intervals (CIs). *P* < 0.05 was considered to be statistically significant. The statistical analyses were performed using the GraphPad Prism 5.

## Supplementary Information

**Additional file 1.** The structured questionnaire.

**Additional file 2.** Original, full-length gel and blot images.

## Data Availability

The data regarding *IL1B*-31C > T genotypes and alleles distributions among participants and the in silico results of software used to support the findings of this study are available from the corresponding author on easonable request.

## Abbreviations

*16S rRNA*: *16 Svedberg ribosomal RNA*
AP-1: Activator protein 1
BDGP: Berkeley Drosophila Genome Project
BLASTn: The nucleotide Basic Local Alignment Search Tool
bosTau3: *Bos Taurus* (cow) full genome as provided by UCSC (Jan 2008)
bp: base pair
C/ EBPβ: CCAAT-enhancer binding protein beta
C/EBPα: CCAAT-enhancer binding protein alpha
canFam2: *Canis lupus familiaris* (domesticated dog) full genome as provided by UCSC (Jan 2008)
CE: Composite regulatory element
ChIP-seq: Chromatin Immunoprecipitation Sequencing
CI: Confidence interval
CS: Composite score
CTPP: Confronting two-pair primer
DR1: Down-regulator of transcription 1
ECR: Evolutionary conserved region
ENCODE: Encyclopedia of DNA elements
FPROM: Human promoter prediction
GATA-1: Globin transcription factor 1
GR: Glucocorticoid receptor
*H. pylori*: *Helicobacter pylori*
hg19: *Homo sapiens* (Human) full genome as provided by UCSC (Feb 2009)
HNF4: Hepatocyte nuclear factor 4
HSF: Heat-shock factor
HSTF: Heat shock transcription factor
HWE: Hardy-Weinberg equilibrium
*IL-1*: *interleukin 1*
*IL1A*: *interleukin 1-alpha*
*IL1B*: *interleukin 1-beta*
*IL-1RN*: *interleukin 1 receptor antagonist*
IRF: Interferon-regulatory factor
LD: Linkage disequilibrium
LDF: Linear discriminant factor
LPS: Lipopolysaccharide
MALT: Mucosa-associated lymphoid tissue lymphoma
MAZ: Myc-associated zinc finger protein
mm9: *Mus musculus* (mouse) full genome as provided by UCSC (Jan 2008)
MSA: Multiple sequence alignment
Mulan: Multiple-sequence local alignment and visualization
NCBI: National Center for Biotechnology Information
NF-AT: Nuclear factor of activated T cells
NF-Y: Nuclear transcription factor Y
NF-κB: Nuclear factor Kappa β
NK: Natural killer cell
NNPP: Neural Network Promoter Prediction
NSAIDS: Non-steroidal anti-inflammatory drugs
Oct-1: Octamer transcription factor
OR: Odds ratio
pan-Tro2: *Pan troglodytes* (chimpanzee) full genome as provided by UCSC (Jan 2008)
PBS: Phosphate buffer saline
PCR: Polymerase chain reaction
PPAR: Peroxisome proliferator-activated receptor
PWM: Position weight matrices
rheMac2: *Macaca mulatta* (rhesus monkey) full genome as provided by UCSC (Jan 2008)
rn4: *Rattus norvegicus* (rat) full genome as provided by UCSC (Jan 2008)
SNP: Single nucleotide polymorphism
Spi-1: SV40 promoter-1
SRF: Serum response factor
SSRP: Structure specific recognition protein 1
STAT1: Signal transducer and activator of transcription 1
Std: Standard
T cell: T lymphocyte cell
T: Thymidine
TBP: TATA- binding protein
TF: Transcription factor
TFBS: Transcription factor binding site
TSS: Transcription start site
TSSG: Recognition of human PolII promoter region and start of transcription
TSSW: Recognition of human PolII promoter region and start of transcription
UTR: Unstranslated region
ZTA: The Epstein-Barr virus bZIP transcription factor
