# Supplementary material for: First insights into the molecular basis association between promoter polymorphisms of the IL1B gene and Helicobacter pylori infection in the Sudanese population: computational approach
Source: BMC Microbiol. 2021 Jan 7;21:16. doi: 10.1186/s12866-020-02072-3 (PMC7792167; doi:10.1186/s12866-020-02072-3)
Supplement: Supplementary file 1 — Additional file 1. The structured questionnaire. [file 12866_2020_2072_MOESM1_ESM.docx]

**Data Sheet**

- Demographics and clinical features of participants

-Participant’s number: ……………..

-Age: …….… -gender:………….… -Nationality…………..…..

-Smoking status: Yes No

-Alcohol intake: Yes No

-Any medicines or (NSAID) are being used ………………...……………..……………..

-Main complaint: ………………………………………………………............……….….

-Diagnosis: ……………………………………………………………….………....……..

-In case of cancerous participants:

Anatomical site……………………: Inflammation………… or Atrophy………..…

Histological type…………………………………………………………………………..

- Investigations results of samples:
- H. pylori infection (16Sr RNA detection) Yes No
- *IL-1B* gene polymorphism ………………………………………………………………………………………

Researcher’s signature ……………………………
