# Supplementary material for: First insights into the molecular basis association between promoter polymorphisms of the IL1B gene and Helicobacter pylori infection in the Sudanese population: computational approach
Source: BMC Microbiol. 2021 Jan 7;21:16. doi: 10.1186/s12866-020-02072-3 (PMC7792167; doi:10.1186/s12866-020-02072-3)
Supplement: Supplementary file 2 — Additional file 2. Original, full-length gel and blot images. [file 12866_2020_2072_MOESM2_ESM.docx]

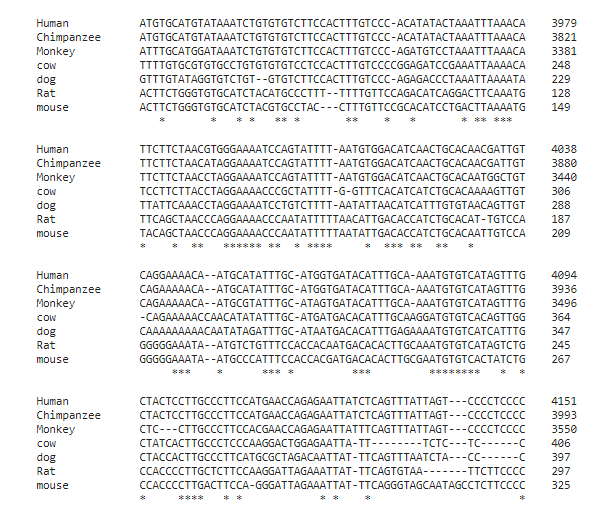

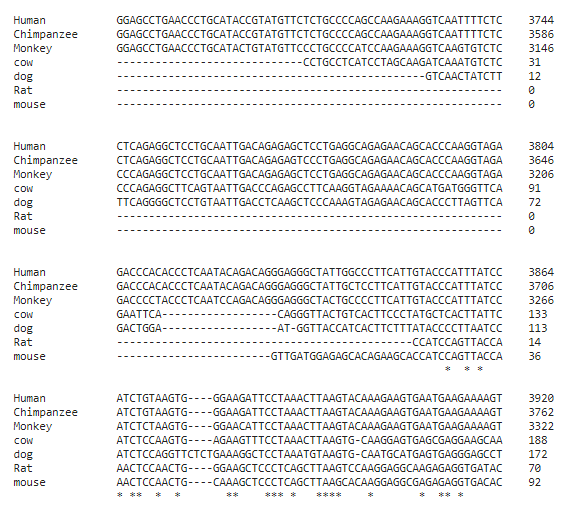


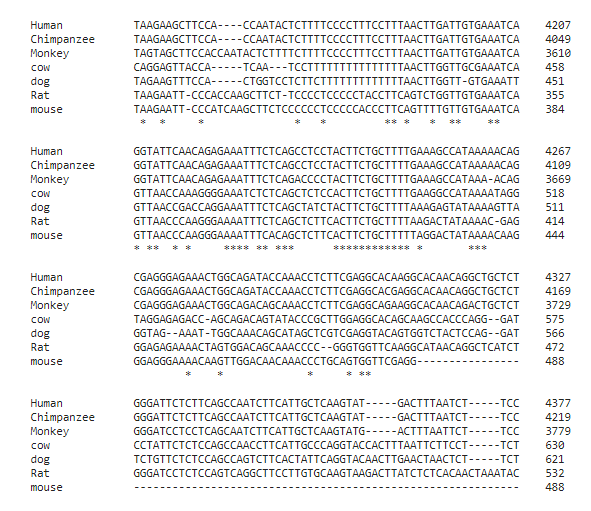


Figure 1. Overview on the mammalian conservation of the IL1B 5′-region [-542_+80] among various species.


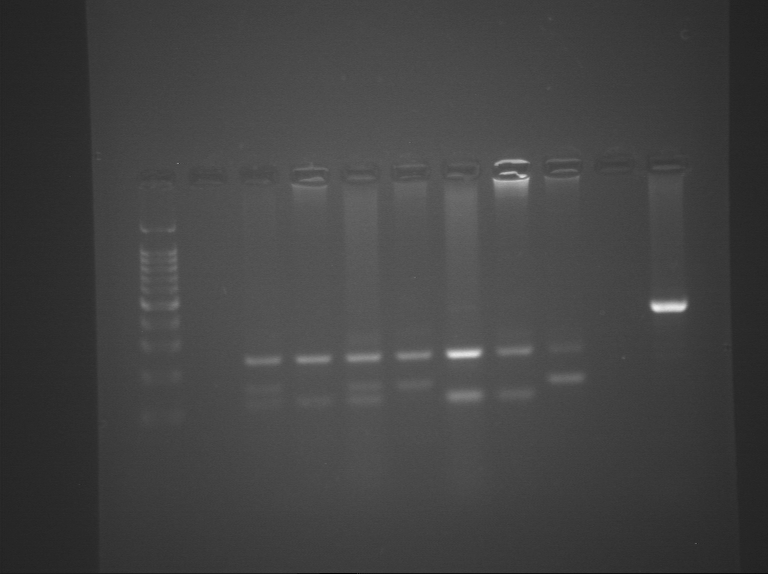


Figure 2. Illustrates PCR-CTPP products analyzed on a 2% agarose gel stained with ethidium bromide. 1and 3 show 240bp, 155bp and 122bp which indicate a heterozygous genotype. 2, 5 and 6 show 240bp and 122bp which indicate a homozygous T genotype. While 4 and 7 show 240bp and 155bp which indicate a homozygous C genotype.


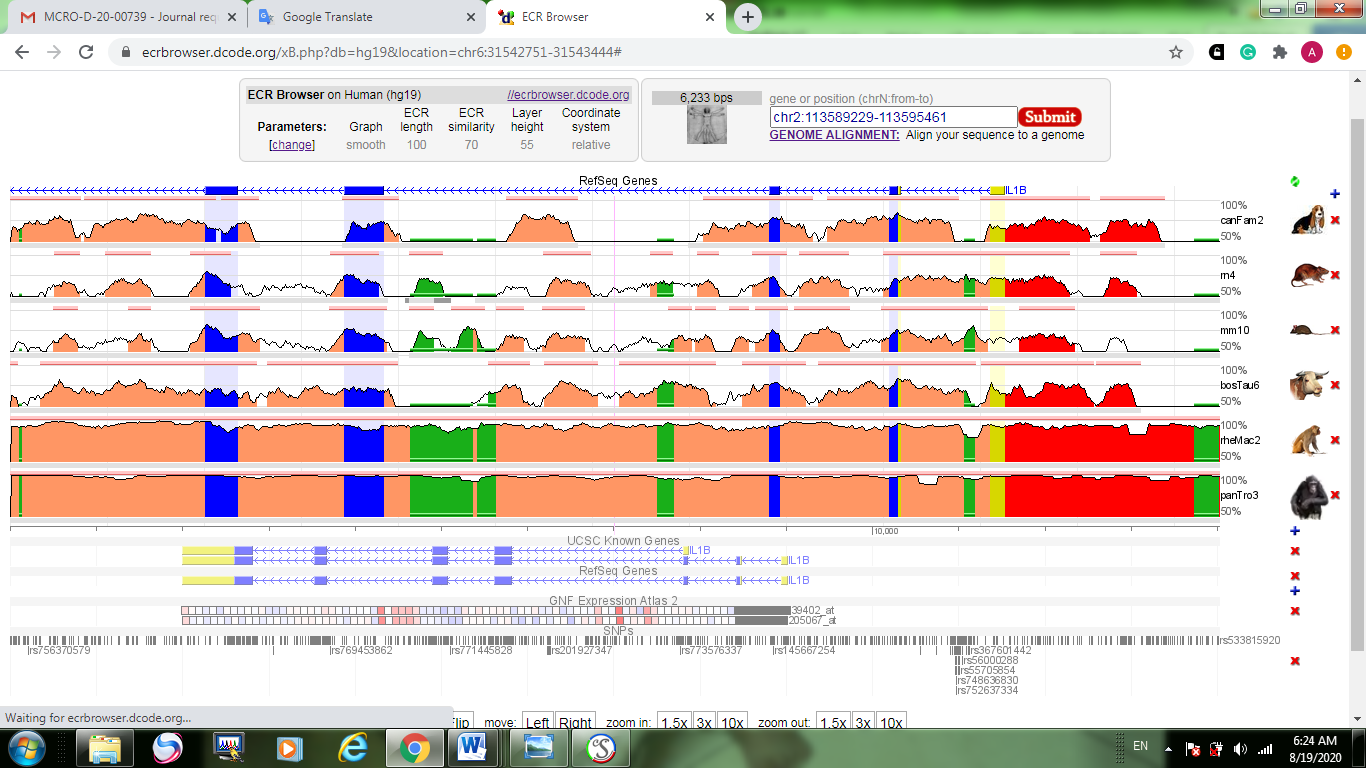


Figure 3. Shows conservation of the *IL1B* 5′-upstream region compared to the human sequence in region [−687_+297] (hg19 chr2:113594193-113594801). The height of the conservation plot at each position represents the number of nucleotides conserved in a windows of 100 nucleotides centered on that position. The pink rectangles at the top of the plot represent the evolutionary conserved regions, which defined as regions of 100 nucleotides with at least 70% identity. Blue boxes represent *IL1B* exon , while yellow indicates the *IL1B* 5′-UTR. Intragenic positions are highlighted in red, or in green when corresponding to transposable elements and simple repeats. Overview from the ECR Browser.


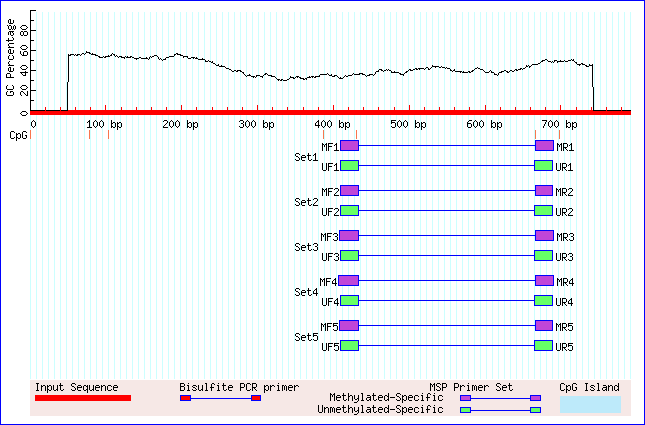


Figure 2. MethPrimer software prediction of no CpG islands in the predicted promoter regions which are located at -328 bp, -124 bp and +1 bp.
